# Responding to and managing antibiotic shortages: a qualitative study with experts and opinion leaders

**DOI:** 10.12688/wellcomeopenres.24292.2

**Published:** 2025-12-26

**Authors:** Vrinda Nampoothiri, Ritika Kondel Bhandari, Avaneesh Kumar Pandey, Oluchi Mbamalu, Jennifer Cohn, Sanjeev Singh, Marc Mendelson, Nusrat Shafiq, Esmita Charani

**Affiliations:** 1 Department of Infection Control and Epidemiology, Amrita Institute of Medical Sciences, Kochi, Kerala, India; 2 Clinical Pharmacology Unit, Post Graduate Institute of Medical Education and Research, Chandigarh, Chandigarh, India; 3 Division of Infectious Diseases and HIV Medicine, University of Cape Town Department of Medicine, Groote Schuur Hospital, Observatory, Western Cape, South Africa; 4 Global Antibiotic Research and Development Partnership, Geneva, Geneva, Switzerland; 5 Faculty of Health Sciences, University of Liverpool Department of Pharmacology and Therapeutics, Liverpool, England, UK

**Keywords:** antibiotic shortages, PESTELI, antibiotic resistance

## Abstract

**Background:**

We investigated the main drivers for current shortages of licensed antibiotics, existing mitigation strategies undertaken and potential barriers in their implementation through in-depth interviews with experts operating in select regions.

**Methods:**

Purposive sampling was used to identify opinion leaders with expertise in managing antibiotic supply chains, access, and shortages between August 2023 and April 2024. Consented participants were interviewed using a semi-structured interview guide developed using the PESTELI (political, economic, sociological, ecological, technological, legal, industry) framework. Data collection and analysis were iterative and recursive, using constant comparison.

**Results:**

Participants who were interviewed (n=16) had local, national, and global roles in managing and studying access, supply, and demand chain management from Europe, South Africa, India and USA. Political engagement on antibiotic shortages is reported to facilitate effective mitigation strategies, especially in areas where there is strong evidence of government investment. Legal measures have also been used; for example, pharmacists in the UK being given rights to automatically substitute antibiotics on prescriptions and negotiating with pharmaceutical companies for greater transparency on the challenges in manufacturing. Economic incentives are currently missing and are recognised as being a driver for lack of engagement on this topic from pharmaceutical industry. Greater transparency is required from the pharmaceutical companies on the manufacturing chain issues that may lead to shortages. Technologically there is a major gap in systems to forecast and manage antibiotic shortages. Sociological elements include adopting appropriate communication to not cause panic buying and hoarding by organisations when there is an impending shortage. Legislative changes are linked to the political and economic barriers for cohesive systems to manage the antibiotic manufacture and supply chain in relation to shortages.

**Conclusion:**

Currently there are limited interventions to respond to and manage shortages. The antibiotic manufacture and supply chain is complex and under the influence of PESTELI indicators which will need to be understood and mitigated in different contexts and regions.

## Plain language summary

Lack of access to licensed antibiotics has been identified as a contributor to the emergence of antibiotic resistance. The drivers for antibiotic shortages are complex. We conducted interviews of key stakeholders (n=16) involved in national and global antibiotic supply chain management, to explore the main drivers for current shortages and existing mitigation strategies undertaken in select regions. Participants were interviewed using a semi-structured interview guide developed using the PESTELI (political, economic, sociological, ecological, technological, legal, industry) framework. Political engagement on antibiotic shortages was reported to facilitate effective mitigation strategies. Legal measures have also been used; for example, pharmacists in the UK given rights to use alternative agents. Pharmaceutical companies need to provide greater transparency on manufacturing issues that may lead to shortages. Economic incentives are required at all levels and are currently missing. Technological solutions to predict antibiotic shortages are needed. From a sociological perspective the response to shortages needs to adopt appropriate communication to not cause panic buying and hoarding by organisations and individuals when there is an impending shortage. Understanding context specific factors contributing to antibiotic shortages can aid in the development and adoption of mitigation strategies and tools for forecasting shortages and their optimised management.

## Introduction

Lack of antibiotic access and excess use remain important factors when considering the emergence and spread of bacterial antimicrobial resistance (AMR) ^1– 3^. Optimising antibiotic use remains a priority in efforts to mitigate AMR, with countries agreeing to a series of commitments to reduce inappropriate antibiotic use at the second United Nations High Level Meeting on AMR in 2024 ^4^. Whilst evidence shows that there has been a 46% increase in the consumption of antibiotics worldwide from 2000 to 2018, lack of access to antibiotics when needed is a threat to safe and effective healthcare delivery particularly in populations in low- and middle-income countries (LMICs) ^5^. Furthermore, whilst the lack of new products in the antibiotic development pipeline is of global concern ^6^ developing new antibiotics alone will not be enough. The lack of market incentives for new antibiotics is influencing the dry pipeline, and managing AMR is further compromised by the inequities in access to new agents, whereby populations in high income countries (HICs) often have easier and faster access than those in LMICs ^7, 8^. Many new antibiotics approved between 2010 and 2020 are still not registered in the majority of LMICs, thereby limiting treatment options for antibiotic-resistant infections ^7^. One reason could be the lack of sufficient local epidemiology data relating to bacterial resistance and antibiotic use. This challenges national programs in prioritising the introduction of new antibiotics or developing accurate forecasting models to predict demand, undermining efforts to support registration of new antibiotics.

In view of the increasing antibiotic shortages, maintaining sustainable supply chains of quality-assured antibiotics is an objective of many AMR national action plans ^9, 10^. The drivers for antibiotic shortages are complex. Low economic returns and/or limited size of the antibiotic manufacturing market have led to fewer supply chain actors. Manufacturing bottlenecks related to the migration of key producers of active pharmaceutical ingredients (API) to single or limited number of suppliers in India and China have further compromised this shrinking market ^11^. At country level there are also regulatory and economic disincentives and lack of political engagement ^11^.

Responding to and managing antibiotic shortages requires a multifocal approach that includes regulatory, production, and supply chain measures. There also needs to be global co-operation that considers the contextual regulatory and economic environment of individual countries whilst managing a global shortages crisis ^12^. Antibiotic shortages can adversely impact the emergence and spread of AMR through the use of alternative broader spectrum antibiotics or those less effective, which may result in higher costs and greater side effects ^13^. Our recent systematic review on the causes and consequences of antibiotic shortages included 74 methodologically diverse studies ^14^. The limited available evidence reports increased length of hospital stay and treatment failure due to using inferior alternative antibiotics during a shortage, and a negative impact on antimicrobial stewardship programmes (AMS), which must realign to the immediate impact of the shortages on institutional antibiotic prescribing patterns and infection management. There is no robust evidence linking shortages to AMR outcomes mainly due to lack of appropriately designed longitudinal studies. Where evidence of impact exists, it is predominantly from HICs (82.4%), indicating a large gap in the literature on the extent of and management of shortages in LMICs. Successful mitigation strategies reported in this review were driven by hospital AMS and infectious diseases teams. In this review, we focused on the USA, where most of the current evidence is generated, Europe, India, and South Africa, two countries with high antibiotic access and AMR challenges ^14^.

To explore the main drivers for current shortages of licensed antibiotics and existing mitigation strategies undertaken in different regions and potential barriers in their implementation, a situational analysis using key informant interviews with actors actively involved nationally and globally in antibiotic supply chain management was conducted. Considering the complexity of the actors and processes in antibiotic manufacturing and supply chains, we adopted a PESTELI (political, economic, sociological, ecological, technological, legal, industry) framework to provide a comprehensive overview and to understand the macro- environmental factors influencing shortages of licensed antibiotics and the ensuing mitigation strategies ^15, 16^.

## Methods

### Study design and data collection

Potential participants were purposively selected to identify those with expertise in antibiotic access and supply chains in HICs and LMICs. Experts were invited to participate in the study through email. Participation in the interview was voluntary. The global, contacted via email had roles at national and regional level across South Africa, Europe, India and USA. Those who agreed to be interviewed were recruited to the study. Written informed consent was obtained from all participants prior to the scheduled interviews. A piloted interview guide was used to conduct interviews face-to-face or on Zoom online platform. The interview guide was developed using the PESTELI framework. The interviews were scheduled at a date and time convenient for the participants. All the interviews were audio-recorded and transcribed verbatim. Due to the lack of evidence from the systematic review on the ecological impact of antibiotic shortages we did not explore this domain and allowed for the study participants to bring this up in the conversation. The study instruments, including interview guide, participant information leaflet and sample consent form are available online on Zivahub ^17^.

### Data analysis

Anonymised transcripts were thematically analysed aided by NVivo 12 software. A coding framework was created after analysing the first five transcripts and was refined by the authors as the coding progressed. Four researchers analysed the interviews independently of each other. Through regular meetings the analysis and framework were discussed and any questions regarding emerging themes were addressed. Data collection was iterative and recursive until thematic saturation was reached.

## Results

Between August 2023 and April 2024, 16 opinion leaders with national, regional, and global roles in antibiotic supply chain management were interviewed. Table 1 gives the details of participants.

**Table 1.** Details of interview participants analysing the current landscape in antibiotic access and supply.

Participant ID	Country	Years in practice	Gender	Sector of work	Scope of work and expertise
A	South Africa	20	M	Global Organisation	Global supply chain management
B	South Africa	20	M	Public Health Sector Regional and National	Global supply chain management
C	South Africa	31	F	Clinical Academic, Public Health Sector and Policy Non-Governmental Organisation	Policy and Health, Access to medicines, including antibiotics
D	Sweden	25	M	Academia and Industry Regional – European Region	Access to medicines, including antibiotics
E	European Union	3	M	Academia and Industry Regional – European Region	Innovation and access of medicines, including antibiotics in the European Union
F	South Africa	36	F	Public Sector and Industry National	Antimicrobial stewardship and antibiotic shortages and access management
G	South Africa	43	M	Academic National Committee	Pharmacology and Pharmacovigilance and National level mitigation of drug shortages
H	South Africa	-	F	Clinical Academic Public Health Sector	Antimicrobial stewardship and access
I	South Africa	-	M	Non-Governmental Organisation National and Regional	Management of availability and shortages of medicines
J	South Africa	-	F	Provincial Health Management Public Health Sector	Hospital Pharmacy Management
K	UK	30	M	National Public Health and Policy	Management of UK supply chain, AMR, antimicrobial stewardship
L	Sweden	20	M	Clinical Academic linked to Industry, Public Health and Policy National and Regional- European Region	Researcher working in the field of AMR and antibiotic access
M	UK	17	M	National Public Health and Policy	Antibiotic prescribing and dose optimisation in field of AMR, Medicine optimization
N	US	8	M	National Public Health and Policy	Experience on developing standards for ensuring quality of medicines, policy levels, Supply chain management
O	UK	-	M	National Public Health and Policy	Economic burden of antimicrobial resistance
P	India	15	M	Academia and Industry National and Regional	New Entry Antibiotics Deal in patent filing from industry and academia, introduction of new antibiotics to the market Supply Chains

### Characteristics of antibiotic manufacture and procurement in different countries

One of the key factors that emerged was differences in antibiotic procurement models between countries and how this may impact the extent of and response to antibiotic shortages. India together with China is one of the main manufacturers and suppliers of antibiotic APIs, a duopoly that can impact global API availability. There is no global procurement model, with countries having their own procurement strategies and processes for all medicines. There are also differences between private and public sectors within countries and by region.

Participants considered pooled procurement across countries as a possible solution to manage the supply of antibiotics wherein countries co-operate to decide on a portfolio of antibiotics and other medications, which would allow them to negotiate a fair market price with the manufacturers (Q1, Table available online on Zivahub ^17^). There was also a recognition of the need for greater transparency in the antibiotic manufacturing and supply chain by pharmaceutical companies (Q2, online Table) ^17^. As the clinical experience of the participants were from South Africa, India, UK, Sweden and the USA, we use these five countries procurement systems as case-studies in managing and responding to shortages of licensed antibiotics and their short-term and long-term impacts Table 2.

**Table 2.** Summary of current systems related to antibiotic shortages including health system, procurement, alert and response systems in the countries of included in this study.

Country	Import versus export index of antibiotics	Public versus private proportion of healthcare	Existing procurement strategies e.g. whether national or regional	Policy and governance in place around managing shortages	Current nationally available data resources on status of or respond to shortages
India	Export more than import (one of the largest producers of antibiotics)	Public sector hospitals account for 10% of hospitals in the country	Procurement occurs at national, federal, state, local government and autonomous body level. Contractual conditions vary and manufacturers bid for contracts at the various levels	Shortages in hospitals in case of outbreaks are often managed by the private pharmacies.	Currently, the shortages are only reported through a website Organisation of Pharmaceutical producers of India (OPPI). However, there is lack of data harmonisation across manufactures and hospitals, which can effectively lead to mitigation of shortages reported
South Africa	Import more that export	Public sector serves 84% of the population	Provincial-level tender system, whereby the price of medicines is set and complied to in the tender. Secondary suppliers fill the gaps in drug stock outs	Locally hospitals develop policies and manage shortages as part of stewardship programmes. In the Western Cape province, the provincial offices respond to and manage shortages by collaborating with specialist groups within their provincial AMS committee to develop alternative therapies. Ledgers are maintained to keep three-month stockholding for the antibiotics as their uptake volume is large. When options are exhausted, public hospitals (which cater to majority of the population) reach out to the private sector.	There is a lack of a national database or antibiotic shortages reporting system in the country
Sweden	Import more than export	Healthcare in Sweden is decentralised, 100% public sector, lies with the regional councils and, in some cases, local councils or municipal governments	There are few pharmaceutical companies that sell generic antibiotics with one company responsible for more than 50% of market sales. In 2023, the Government commissioned the Public Health Agency of Sweden to carry out a preliminary study of a new reimbursement model where pharmaceutical companies receive compensation for providing a buffer stock of certain prioritised off- patent antibiotics	Three agencies are involved in governance to manage shortages: reimbursement agency, Public Health Agency, the Medical Product Agency, equivalent to the USA Food and Drug Agency (FDA) or European Medicines Agency (EMA). Among these, the Public Health Agency has a unique role in engaging openly with the industry regarding shortages. They organize workshops, facilitate discussions, and have spearheaded innovative reimbursement models	The Public Health Agency of Sweden (PHAS) of Sweden has established policies for managing antibiotic shortages. PHAS has been tasked with proposing and piloting models to ensure the availability of approved antibiotics, aiming to combat antibiotic resistance as part of a broader public health strategy
UK	Import more than export	The UK has a 100% public sector by government- sponsored universal healthcare system called the National Health Service (NHS). Private sector healthcare system is voluntary for rapid access to elective care and other services.	the Department of Health and Social Care (DHSC) is responsible for ensuring a stable supply of antibiotics across the country. For patented branded medicines, pharmaceutical companies agree a fixed price with manufacturers to secure supply. For generic antibiotics, however, the supply chain is managed through three distinct contracted areas based on geographic regions, with two-year rolling cycles to maintain consistent availability across the nation.	In case of shortages, Department of Health in the UK leads on supply chain for primary care, whereas NHS England work for leads on supply chain for hospitals.	There is a national dashboard in Department of Health and social care where upcoming shortages and stock-outs are reported. The Department releases guidance on national shortage antibiotics to advise on alternative therapies
USA	Import more than export	Hospitals are largely (80%) operated in the private sector	Subscription Models involve fixed payments to pharmaceutical companies for access to antibiotics, which may include provisions for both fixed and volume- based components of payment. Pooled Procurement Models consolidates purchasing power among healthcare providers or systems to negotiate better prices and terms for antibiotics.	There is an essential medicine list from which vulnerable medicines (those at increased risk of being affected by shortages) are selected for local manufacture. Some key reasons to categorise a medicine as vulnerable include geographic concentration, complexity of manufacturing the medicine, quality testing issues, and price.	The FDA gathers data on medicine shortages in the country including antibiotics. The Academy of Health Systems Pharmacists hosts a resource page for drug shortages, with self-reported data by pharmacists. As of 30 September 2024, there were 41 active antimicrobial shortages reported in the USA. The data on these resources, however, are limited to notice of a shortage and an expected end date. There are no data on policy or formulary change recommendations, mitigation strategies at healthcare system level or a report of consequences of these shortages.

### Causes of antibiotic shortages

**Figure 1.**
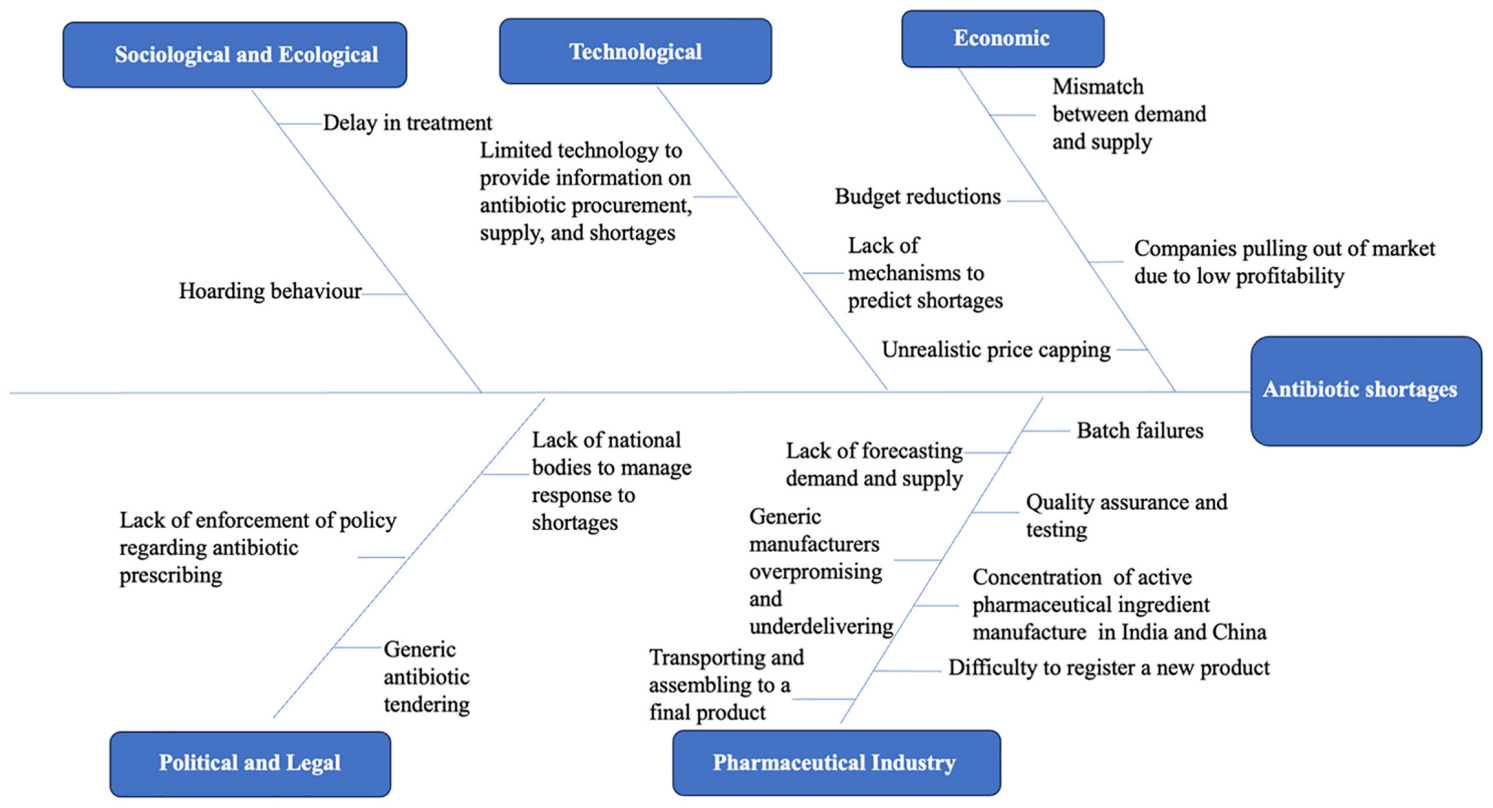
Key causes of antibiotic shortages as reported by the study participants mapped to the political, economic, sociological and ecological, technological, legal, and pharma industry (PESTELI) framework.

Causes for antibiotic shortages reported by study participants ranged from concentration of API manufacturing in only a few countries to lack of adequate forecasting from manufacturers and lack of strict regulations at government level. The key causes for antibiotic shortages as informed by the interview participants is illustrated in Figure 1.

The individual elements of the PESTELI framework which play roles in the antibiotic manufacturing, procurement and management of antibiotic shortages which were discussed during the interviews are described in the sections below.

### Political, policy, and legal frameworks within context of countries

Participants agreed that prioritisation of antibiotic shortages by governments is beneficial especially in developing and implementing guidelines for alternative antibiotics (Q3, online table) ^17^. Participants from the UK identified the Department of Health and the NHS leadership as playing a role in managing shortages and that there are communication mechanisms between them and subject matter experts to assess the impact of shortages on clinical and patient outcomes (Q4, online table) ^17^. In Sweden, there are government-assigned authorities, such as the Medical Products Agency, that work on antibiotic availability and develop strategies to mitigate any immediate or future shortages (Q5, online table) ^17^. However, the lack of effective and easily accessible communication from the government has been highlighted by participants from South Africa (Q6, online table) ^17^.

As only China and India predominantly control API supply, participants considered that during shortages API supply to countries that produce higher profit margins may be prioritized, to the disadvantage of other countries. Additionally, incorrect national estimates and delayed provincial payments hinder the ability to maintain sufficient buffer stock, exacerbating supply issues. In South Africa participants highlighted that shortages may be caused by contractual transition between suppliers where one supplier scales down and does not fulfil their orders towards the end of their contract, while the new supplier takes longer to begin production (Q7, online table) ^17^. In the USA, whilst government is invested in supporting gathering of basic data on medicines shortages, current resources do not allow for sufficient data to be gathered that can help better understand the causes and consequences to better inform policy and practice (Q8, online table) ^17^.

### Economic factors

Participants agreed that the pricing of antibiotics plays a role in shortages. Price capping is considered not viable, incompatible with current market prices, and a potential driver of shortages (Q9, online table) ^17^. Successive budget cuts in hospitals can also impact the stocks held in the pharmacies, reducing the buffer stock that could have prevented shortages (Q10, online table) ^17^. Optimal use of the manufacturing capacity is important since limited use of capacity can result in increased antibiotic production costs (Q11, online table) ^17^.

Due to the low profitability of the market and low return of investments, suppliers are pulling out of market which could result in antibiotic shortages especially if there is no innovator brand (Q12, online table) ^17^. When prices are low, drug sales need to increase to maintain profitability. Having only a few suppliers producing large volumes of an antibiotic can also lead to shortages (Q13, online table) ^17^. Low profit margin for generic antibiotics is a market force driving lack of investment. The high pressure on lowering the cost of generic antibiotics has resulted in increasing numbers of generic manufacturers leaving the field or focusing on those antibiotics which remain profitable (Q14, online table) ^17^. In the European Union, pull incentives schemes such as the Swedish model aims to generate additional manufacturer revenue to maintain the supply chain of five selected antimicrobials. (Q15, online table) ^17^. The minimal economic incentives for pharmaceutical industry to manufacture antibiotics has resulted in financial strains for many companies. They are also disincentivised to sell antibiotics in those countries where they would lose money selling antibiotics.

Solutions to managing shortages may also have economic consequences. A potential economic approach that may offer insights is the US Presidency Emergency Plan for AIDS Relief (PEPFAR) model which supports countries to locally produce antiretrovirals, expanding local pharmaceutical production capabilities as well as procurement ^18^. This option, however, relies on NGO commitment, is costly to governments, and may be therefore have its limitations. The other model is when there is an intermediary between manufacturers and national procurers who purchases the drugs and sells them (Q16, online table) ^17^.

### Sociological factors

Antibiotic shortages result in delays in treatment, in addition to patients having to purchase antibiotics with higher costs and adverse effects (Q17, online table) ^17^. This is of significant consequence to paediatric patients due to limited alternative formulations (Q18, online table) ^17^. These could be contributing factors to the emergence of AMR in the long run. Availability of information to the public regarding antibiotic shortages was considered to have positive and negative consequences in relation to demand for antibiotics. A lack of sufficient information provided to patients on antibiotic use could increase demand for antibiotics, particularly in countries without universal healthcare coverage (Q19, online table) ^17^, whereas in countries with universal healthcare coverage the opposite may occur (Q20, online table) ^17^. Appropriate education on antibiotic use ideally is needed from school age, customised for the different age groups and target population literacy levels (Q21, 22, online table) ^17^. This highlights the differences in populations across and within countries in terms of access to resources that benefit health, and health beliefs and practices.

Participants also informed that hoarding leftover medicines for future use because of difficulty in accessing healthcare may be practiced, particularly from community pharmacies (Q23, online table) ^17^. Social media was recognized as having a role in encouraging the public to hoard medicines especially when there is a shortage (Q24, online table) ^17^. Better communication is also important between suppliers and hospitals to ensure that sufficient stocks are available (Q25, online table) ^17^.

Mitigation strategies in South Africa include moving stocks between facilities with involvement of the pharmacy and therapeutics committee and AMS teams in hospitals to advise prescribers on alternatives (Q26, online table).

### Technological factors

The need for available technology to provide information on antibiotic procurement, supply, and shortages was recognised by participants. It is important to have a mechanism to track indicators of shortages in real time, which can be used to predict shortages that will enable better management. Implementation and adoption of such of technologies is inconsistent. In the UK, communication of antibiotic shortages is such that sufficient information is provided so that the people can be prepared without resulting in increased demand (Q27, online table). The public have access to an app from where they can see the medicine stocks and the nearest pharmacy where they would be available (Q28, online table) ^17^. There is also a bulletin, which provides real-time data on shortages and any supply chain issues wherein the healthcare providers can log in and see how long a shortage is going to last and what are the alternatives available for the medicine in shortage. Additionally, a medicine supply notification system alerts hospital and community pharmacies to shortages providing a description of the shortage along with its durations and available alternatives (Q29, online table) ^17^. Another strategy adopted in the UK was implementing a dashboard to provide live dispensing data thereby enabling forecast of how many days of stock is left (Q30, online table).

In many countries, including Sweden, healthcare institutions do not use automated information systems for procurement and prescribing, leading to a lack of data linkage for information sharing and longitudinal assessment of supply patterns and any interruptions to the supply of medicines including antibiotics (Q31, online table) ^17^. The Medical Product Agency has built a national system to track the availability of antibiotics wherein registered users, predominantly prescribers, can log in and check availability of specific antibiotics. The system however is not widely adopted. In response to this, currently efforts are in place to build a system that will allow prescribers to see the availability of the antibiotic when they prescribe it (Q32, online table) ^17^. Organisations such as Indian Drug Manufacturers Association (IDMA) and Organisation of Pharmaceutical Producers of India (OPPI) enable pharmaceutical manufacturers to register and maintain a consolidated database, which can be accessed upon request during shortages (Q33, online table) ^17^. In South Africa there is a stock visibility system, however, there too the technology fails to link provincial and national databases, and overall, negates any benefit of the alert system (Q34, online table) ^17^. USA has implemented the medicines supply chain map that includes data of manufacturing of all drugs that is being supplied to the USA. This data can indicate and quantify shortages in categories (Q35, online table) ^17^.

### Legal factors and frameworks

Many of the HICs have poorly enforced regulations in place that mandate pharmaceutical companies to alert healthcare systems to the likelihood or risk of upcoming drug shortages. If implemented effectively such legal mandates may lead to more consistent data on antibiotic shortages. Because where such legislation does exist it is predominantly in HICs, this may mean that at an international level, what gets prioritised in terms of response to antibiotic supply issues is often driven by HIC needs, whilst the impact of AMR and antibiotic shortages are invariably more pronounced In LMICs (Q36, online table) ^17^.

In Europe, the European commission is responsible for improving innovation and access to medical countermeasures and is responsible for dealing with disruption the antibiotic supply chain (Q37, online table) ^17^. The ‘serious shortages protocol’, a legal instrument available in the UK allows pharmacists to dispense alternative antibiotics without consulting the prescriber during an antibiotic shortage (Q38, online table) ^17^. The Department of Health and Social Care has legal responsibility for ensuring that medicines are available in the country and that manufacturers report a shortage or expectation of one, directly to the Department of Health (Q39, online table) ^17^. While this system has strengths such as good communication and awareness around antibiotic shortages, its weakness lies in the lack of real-time information on stock holdings and community pharmacies across the country (Q40, online table) ^17^. The USA, Canada and Australia have similar legal requirements, but this may not be the case in LMICs.

### Industry factors

By and large, the role of industry is absent as an active partner in working to mitigate shortages. Hospital pharmacists in South Africa through their supplier networks are likely to be informed of impending antibiotic shortages in an ad-hoc fashion, with no formal mechanism of communication between pharmaceutical manufacturers and the Department of Health (Q41, online table) ^17^. In the UK, there is a legal requirement wherein the manufacturers need to notify the Department of Health in case of any shortages (Q42, online table) ^17^. Monopolisation of API production by a few countries like India and China was reported as a challenge particularly if production is impacted (Q43, online table) ^17^. Participants also reported problems with ensuring the quality of the products received, especially since organisations like the South African Health Product Regulatory Authority (SAHPRA) monitors the testing facilities but does not conduct quality tests of the products (Q44, online table) ^17^.

**Figure 2.**
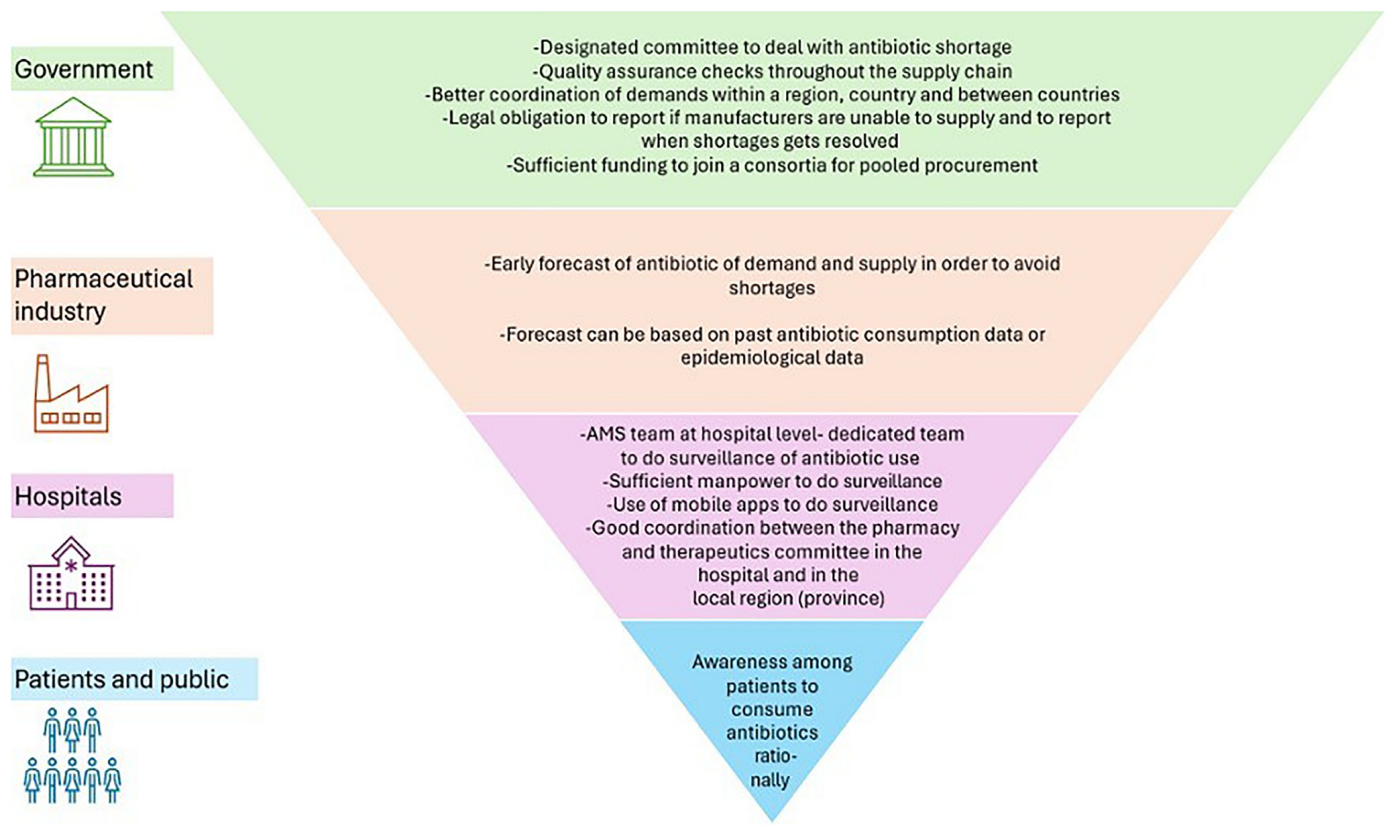
Potential mitigation strategies for forecasting, responding to and managing antibiotics shortages from the macro to meso level.

In addition to the mitigation strategies elaborated in the above sections, participants also provided some potential strategies to better handle situations of antibiotic shortages. We have stratified these by government, pharmaceutical industry, hospital and patient and public sector levels, shown in Figure 2.

## Discussion

This study used key informant interviews to explore the main drivers for current shortages, existing mitigation strategies undertaken in different regions and potential barriers in their implementation using a PESTELI framework. A key contributing factor to antibiotic shortages suggested by the participants was the duopoly of antibiotic supply from India and China, which risks production and global supply chain disruption ^19^. Study participants recommended using a pooled procurement system for antibiotics, which would allow negotiation of a fair price with manufacturers. The systematic review by Parmaksiz *et al*., on pooled procurement of medicines and vaccines recommends that this mechanism can result in lower prices, increased availability, higher quality and more efficient procurement processes ^20^. Berman and colleagues also recommend the adoption of an antimicrobial subscription and pooled procurement model, anchored by regional market leaders and guaranteeing public and private sector access to antimicrobials ^21^. The low profitability of the antibiotics has resulted in suppliers leaving the market. Whilst there are pull incentives in place in many countries, little attention has been paid to finance of these incentives ^22^.

In LMICs, weak laboratory capacity, lack of health information systems, and limited resources results in low quality data, which does not show the actual scenario of AMR in these countries ^23^. In many LMICs, access to antibiotics is poor but inappropriate use reduces supply further. For example, in India, a country with high burden of infectious diseases and poorly regulated access to antibiotics, a lack of community-level antibiotic stewardship leads to excess use ^24^. HICs also have stricter reporting of shortages. Registries for pharmaceutical companies to report upcoming shortages and their estimated duration are a vital tool to enable a quick response from the government ^13^. Government and subject-matter experts in the UK collaborate to assess the impact of shortages on clinical and patient outcomes. Governments also work with pharmaceutical companies to review stockpiling policies to ensure sufficient stocks anticipating imbalances between supply and demand ^25^.

AMS initiatives and effective communication between physicians, pharmacists, and stockists is recommended as an effective strategy to mitigate antibiotic shortages ^12^. Participants in our study also suggested that structured communication should also be provided to patients and public, which could result in a decrease in unnecessary antibiotic demand from their end. Direct patient counselling by healthcare provider and printed education material can reduced antibiotic use ^26^. Whilst the majority of the national action plans emphasise public education as a means for raising awareness on AMR, only limited strategies have been identified in these plans to achieve this awareness ^9^. Age-appropriate education on antibiotic use should be initiated in schools and there should be more funding provided for conducting community engagement activities on AMR especially in LMICs with unregulated access to antibiotics.

### Limitations of the study

We were interested in exploring the antibiotic shortages in India in detail, however, despite inviting many potential participants, only one consented to being interviewed. We reached out to potential participants from the pharmaceutical industries in different countries to contribute to the learning in this study, but due to employment clauses and confidentiality we could not interview them. Despite these limitations, the study provides a detailed presentation of the key factors that play a role in antibiotic shortages in different countries and contexts. The ecological implications of antibiotic shortages did not emerge from these interviews. Based on our existing work and the systematic review we have undertaken this aspect remains unexplored. The migration of major API producers to two countries and the process of manufacturing of antibiotics and treatment of waste materials may have implications for those populations and this is something that needs to be studied.

## Conclusion

This study shows that the causes of antibiotic shortages are multi factorial and requires interdisciplinary mitigation strategies. Currently there are limited interventions to respond to and manage shortages. The antibiotic manufacture and supply chain is complex and under the influence of various PESTELI indicators which will need to be understood and managed in different contexts and regions. Better understanding the complex factors contributing to antibiotic shortages will support development and adoption of mitigation strategies and tools for forecasting shortages and their optimised management. Different actors at the government, healthcare, pharmaceutical industry and public levels should work together to manage antibiotic use and shortages. In addition, it is important that the strategies developed are contextually fit after critically assessing the existing local demand and supply chains within countries and the political, legislative, and economic incentives for effective participation of all actors concerned.

## Ethics approval and consent to participate

Institutional approval to conduct the study was provided by the human research ethics committees (HREC) of the University of Cape Town (UCT), South Africa (UCT HREC: 488/2022) and Amrita Institute of Medical Sciences, Kochi, Kerala, India (IEC-AIMS-2022-MEDADN-164). Written informed consent was obtained from all participants prior to interview.

## Data Availability

The datasets used and/or analysed during the current study, together with the data analysed and table of quotes are available from data repository at University of Cape Town https://doi.org/10.25375/uct.29178665.v4 ^17^ Data are available under the terms of the Creative Commons Attribution 4.0 International license (CC-BY 4.0).
