# Supplementary material for: Responding to and managing antibiotic shortages: a qualitative study with experts and opinion leaders
Source: Wellcome Open Res. 2025 Dec 26;10:347. Originally published 2025 Jul 14. [Version 2] doi: 10.12688/wellcomeopenres.24292.2 (PMC12856250; doi:10.12688/wellcomeopenres.24292.2)
Supplement: Peer review history

# Reviewer report: Reviewer response for version 2

**DOI:** 10.21956/wellcomeopenres.27986.r143189

**Published:** 2026-02-19

**Authors:** Bashar Haruna Gulumbe

**Affiliations:** 1 Department of Microbiology, Federal University Birnin Kebbi, Birnin Kebbi, Kebbi, Nigeria

The authors have addressed my concerns. I have no further comments.

Is the work clearly and accurately presented and does it cite the current literature?

Partly

If applicable, is the statistical analysis and its interpretation appropriate?

Yes

Are all the source data underlying the results available to ensure full reproducibility?

Yes

Is the study design appropriate and is the work technically sound?

Partly

Are the conclusions drawn adequately supported by the results?

Partly

Are sufficient details of methods and analysis provided to allow replication by others?

Yes

Reviewer Expertise:

Antimicrobial resistance, microbial ecology, environmental microbiology, omics, and antimicrobial stewardship.

I confirm that I have read this submission and believe that I have an appropriate level of expertise to confirm that it is of an acceptable scientific standard.

# Reviewer report: Reviewer response for version 2

**DOI:** 10.21956/wellcomeopenres.27986.r143190

**Published:** 2026-01-29

**Authors:** Michael Samarkos

**Affiliations:** 1 National and Kapodistrian University of Athens, Athens, Greece

I have read the revised manuscript and the authors' responses to my comments.

 I am satisfied with the changes made to the manuscript.

Is the work clearly and accurately presented and does it cite the current literature?

Yes

If applicable, is the statistical analysis and its interpretation appropriate?

Not applicable

Are all the source data underlying the results available to ensure full reproducibility?

Yes

Is the study design appropriate and is the work technically sound?

Yes

Are the conclusions drawn adequately supported by the results?

Partly

Are sufficient details of methods and analysis provided to allow replication by others?

Partly

Reviewer Expertise:

Antimicrobial resistance, antimicrobial stewardship

I confirm that I have read this submission and believe that I have an appropriate level of expertise to confirm that it is of an acceptable scientific standard.

# Reviewer report: Reviewer response for version 2

**DOI:** 10.21956/wellcomeopenres.27986.r143191

**Published:** 2026-01-07

**Authors:** Enrico Baraldi

**Affiliations:** 1 Uppsala University, uppsala, Sweden

Thanks for the opportunity to reassess this work. I am pleased to see that the authors have implemented basically all my suggestions. Therefore, from my perspective the paper is now ready for final acceptance.

Is the work clearly and accurately presented and does it cite the current literature?

Yes

If applicable, is the statistical analysis and its interpretation appropriate?

Not applicable

Are all the source data underlying the results available to ensure full reproducibility?

Yes

Is the study design appropriate and is the work technically sound?

Yes

Are the conclusions drawn adequately supported by the results?

Partly

Are sufficient details of methods and analysis provided to allow replication by others?

Yes

Reviewer Expertise:

NA

I confirm that I have read this submission and believe that I have an appropriate level of expertise to confirm that it is of an acceptable scientific standard.

# Reviewer report: Reviewer response for version 1

**DOI:** 10.21956/wellcomeopenres.26790.r128634

**Published:** 2025-09-10

**Authors:** Michael Samarkos

**Affiliations:** 1 National and Kapodistrian University of Athens, Athens, Greece

Responding to and managing antibiotic shortages: a qualitative study with experts and opinion leaders (Wellcome Open Research 2025, 10:347 )

 SUMMARY OF THE ARTICLE

 This is a qualitative research study investigating how different countries respond to and manage antibiotic shortages. It should be made clear that the study is based on interviews with experts in managing antibiotic supply chains, rather than facts collected by the authors themselves.

 The authors used purposive sampling of experts in the field and conducted semi-structured interviews which they analyzed with specialized software. The interviews were structured using the PESTELI (political, economic, sociological, technological, legal, industry) framework, thus the results are similarly provided using the same framework.

 The results are divided into:

- Characteristics of antibiotic manufacture and procurement in different countries
- Causes of antibiotic shortages
- Factors affecting antibiotic manufacturing, procurement and managing antibiotic shortage (reported per individual element of the PESTELI framework)
- Mitigation strategies for antibiotic shortages

The results are generally presented clearly, and the authors provide useful graphical summaries of the key causes of antibiotic shortages and of mitigation strategies.

 SPECIFIC QUESTIONS

- Is the study design appropriate and is the work technically sound?
  - Yes
- Are sufficient details of methods and analysis provided to allow replication by others?
  - Partly. There is room for improvement, especially regarding the sampling process: The authors should provide details for the recruitment rationale and for the experts mix, report invitations to experts, non-responses etc.
  - Providing a thematic saturation grid might be useful.
  - I would suggest that the authors provide information on study conformation with the existing standards for reporting qualitative research (SRQR, COREQ etc)
- If applicable, is the statistical analysis and its interpretation appropriate?
  - Not applicable.
- Are all the source data underlying the results available to ensure full reproducibility?
  - Yes
- Are the conclusions drawn adequately supported by the results?
  - Mostly yes, but key findings should be set out more clearly.

COMMENTS

 In the introduction the authors stress the role of antibiotic shortages in AMR development, however they offer few insights into this issue thereafter. In the Results section, AMR is only mentioned once (in the Legal factors subsection).

 The title of the manuscript refers to “responses and management of antibiotic shortages”, but in the last paragraph of the Introduction the authors also state that “exploring the main drivers” was also an aim of the study. Exploring the drivers of shortages themselves is quite complex, as the causes (and the management) differ depending on the level of shortages i.e., local, national or global. It, therefore, seems to me that this study has too broad a scope. I am not sure that all these issues can be covered adequately. I suggest that the authors focus more on national or global shortages.

 The breadth of scope may cause issues. E.g. in p 8, subsection “Sociological factors” the authors initially discuss **some effects** of antibiotic shortages (“Antibiotic shortages result in delays in treatment, in addition to patients having to purchase antibiotics etc”), which is not relevant there.

 Participants imbalance is an important issue. 8/16 experts are from South Africa, while there is only one expert from India and none from China (which are the two major producers of API). This creates imbalance in the results as e.g. in South Africa 84% of healthcare is covered by the public sector. Consequently, although thematic saturation might have been reached, the responses would cover much more the problems of the public rather than private sector. The authors acknowledged some but not all of these issues in the limitations (e.g not explanation for the lack of participants from China).

Is the work clearly and accurately presented and does it cite the current literature?

Yes

If applicable, is the statistical analysis and its interpretation appropriate?

Not applicable

Are all the source data underlying the results available to ensure full reproducibility?

Yes

Is the study design appropriate and is the work technically sound?

Yes

Are the conclusions drawn adequately supported by the results?

Partly

Are sufficient details of methods and analysis provided to allow replication by others?

Partly

Reviewer Expertise:

Antimicrobial resistance, antimicrobial stewardship

I confirm that I have read this submission and believe that I have an appropriate level of expertise to confirm that it is of an acceptable scientific standard, however I have significant reservations, as outlined above.

# Response

**Published:** 2025-11-25

**Authors:** ESMITA CHARANI

**Affiliations:** Medicine, University of Cape Town, Cape Town, Western Cape, South Africa

**Comment: **This is a qualitative research study investigating how different countries respond to and manage antibiotic shortages. It should be made clear that the study is based on interviews with experts in managing antibiotic supply chains, rather than facts collected by the authors themselves.

 The authors used purposive sampling of experts in the field and conducted semi-structured interviews which they analyzed with specialized software. The interviews were structured using the PESTELI (political, economic, sociological, technological, legal, industry) framework, thus the results are similarly provided using the same framework.

 The results are divided into:

- Characteristics of antibiotic manufacture and procurement in different countries
- Causes of antibiotic shortages
- Factors affecting antibiotic manufacturing, procurement and managing antibiotic shortage (reported per individual element of the PESTELI framework)
- Mitigation strategies for antibiotic shortages

The results are generally presented clearly, and the authors provide useful graphical summaries of the key causes of antibiotic shortages and of mitigation strategies.     Response The authors thank the reviewer for highlighting the relevance of the study.   Comment Specific questions: Are sufficient details of methods and analysis provided to allow replication by others?

- Partly. There is room for improvement, especially regarding the sampling process: The authors should provide details for the recruitment rationale and for the experts mix, report invitations to experts, non-responses etc.
- Providing a thematic saturation grid might be useful.
- I would suggest that the authors provide information on study conformation with the existing standards for reporting qualitative research (SRQR, COREQ etc)

**Response: **For qualitative research we do not usually provide non-respondent data. We have now provided the rationale for the recruitment and the limitations section does provide details on the implications of this strategy.    

**Comment: **In the introduction the authors stress the role of antibiotic shortages in AMR development, however they offer few insights into this issue thereafter. In the Results section, AMR is only mentioned once (in the Legal factors subsection).

 The title of the manuscript refers to “responses and management of antibiotic shortages”, but in the last paragraph of the Introduction the authors also state that “exploring the main drivers” was also an aim of the study. Exploring the drivers of shortages themselves is quite complex, as the causes (and the management) differ depending on the level of shortages i.e., local, national or global. It, therefore, seems to me that this study has too broad a scope. I am not sure that all these issues can be covered adequately. I suggest that the authors focus more on national or global shortages.  

**Response: **The aim of the paper has been modified to a situational analysis using  key informant interviews with actors actively involved, nationally and globally, in antibiotic supply chain management to explore the main drivers for current shortages, existing mitigation strategies undertaken in different regions and potential barriers in their implementation.  

**Comment: **The breadth of scope may cause issues. E.g. in p 8, subsection “Sociological factors” the authors initially discuss **some effects** of antibiotic shortages (“Antibiotic shortages result in delays in treatment, in addition to patients having to purchase antibiotics etc”), which is not relevant there.  

**Response: **We had added this point to show link between antibiotic shortages and AMR especially in pediatric population. We have modified the point to further clarify this (line 344)  

**Comment: **Participants imbalance is an important issue. 8/16 experts are from South Africa, while there is only one expert from India and none from China (which are the two major producers of API). This creates imbalance in the results as e.g. in South Africa 84% of healthcare is covered by the public sector. Consequently, although thematic saturation might have been reached, the responses would cover much more the problems of the public rather than private sector. The authors acknowledged some but not all of these issues in the limitations (e.g not explanation for the lack of participants from China).  

**Response: **Participation in the interview was voluntary. We reached out to stakeholders in South Africa, Europe, India and USA and interviewed those participants who consented.

# Reviewer report: Reviewer response for version 1

**DOI:** 10.21956/wellcomeopenres.26790.r129884

**Published:** 2025-08-30

**Authors:** Bashar Haruna Gulumbe

**Affiliations:** 1 Department of Microbiology, Federal University Birnin Kebbi, Birnin Kebbi, Kebbi, Nigeria

This paper seeks to address the important topic of antibiotic shortages and the challenges they create for healthcare systems and antimicrobial stewardship. The authors explore these issues using expert interviews framed through the PESTELI lens. The subject matter is highly relevant, and the need to better understand supply chain vulnerabilities is clear. However, after careful consideration, I have significant concerns about the strength of the evidence and the scope of the conclusions that are drawn as follows:

1. The study does not directly contribute to our understanding of the drivers of antimicrobial resistance. Shortages may certainly contribute indirectly, through inappropriate substitutions or the use of broader spectrum agents, but this study does not present empirical data linking shortages to resistance outcomes. The framing of shortages as a driver of resistance is therefore stronger than the evidence allows.
2. The evidence base is also limited. Sixteen expert interviews across five countries provide useful perspectives, but the sample is heavily weighted toward South Africa and high income settings. Large regions such as Latin America, much of Africa, and Southeast Asia are missing, and perspectives from the pharmaceutical industry are largely absent. This limits the ability to generalise the findings or present them as a global picture.
3. The PESTELI framework offers structure for analysis, but the study remains descriptive and exploratory. Some of the conclusions read as prescriptive and definitive when they should be framed more cautiously. In addition, the ecological dimension of shortages was not explored, despite its importance in antibiotic manufacturing and the potential link to antimicrobial resistance in the environment.
4. Another issue is novelty. The authors recently published a systematic review on antibiotic shortages, and the present work risks being seen as incremental rather than substantially new. The overlap between the two outputs should be made very clear.

On balance, I believe the paper has potential value as a situational analysis of supply chain perspectives, but in its current form it does not make a sufficiently strong or novel contribution to merit indexing.

Is the work clearly and accurately presented and does it cite the current literature?

Partly

If applicable, is the statistical analysis and its interpretation appropriate?

Yes

Are all the source data underlying the results available to ensure full reproducibility?

Yes

Is the study design appropriate and is the work technically sound?

Partly

Are the conclusions drawn adequately supported by the results?

Partly

Are sufficient details of methods and analysis provided to allow replication by others?

Yes

Reviewer Expertise:

Antimicrobial resistance, microbial ecology, environmental microbiology, omics, and antimicrobial stewardship.

I confirm that I have read this submission and believe that I have an appropriate level of expertise to state that I do not consider it to be of an acceptable scientific standard, for reasons outlined above.

# Response

**Published:** 2025-11-25

**Authors:** ESMITA CHARANI

**Affiliations:** Medicine, University of Cape Town, Cape Town, Western Cape, South Africa

**Comment: **This paper seeks to address the important topic of antibiotic shortages and the challenges they create for healthcare systems and antimicrobial stewardship. The authors explore these issues using expert interviews framed through the PESTELI lens. The subject matter is highly relevant, and the need to better understand supply chain vulnerabilities is clear. However, after careful consideration, I have significant concerns about the strength of the evidence and the scope of the conclusions that are drawn as follows:

**Response: **We have responded to your comments below.  

**Comment: **The study does not directly contribute to our understanding of the drivers of antimicrobial resistance. Shortages may certainly contribute indirectly, through inappropriate substitutions or the use of broader spectrum agents, but this study does not present empirical data linking shortages to resistance outcomes. The framing of shortages as a driver of resistance is therefore stronger than the evidence allows.  

**Response: **The aim of the study was to conduct a situational analysis using key informant interviews with actors actively involved, nationally and globally, in antibiotic supply chain management to explore the main drivers for current shortages, existing mitigation strategies undertaken in different regions and potential barriers in their implementation. We intended to show the complexity of the issue and highlight the need for an interdisciplinary approach to mitigate antibiotic shortages.  

**Comment: **The evidence base is also limited. Sixteen expert interviews across five countries provide useful perspectives, but the sample is heavily weighted toward South Africa and high-income settings. Large regions such as Latin America, much of Africa, and Southeast Asia are missing, and perspectives from the pharmaceutical industry are largely absent. This limits the ability to generalise the findings or present them as a global picture.  

**Response: **As mentioned in our limitation section, more participants were approached but due to lack of consent, we could not interview them. This was not a global study with participants from all countries.  

**Comment: **The PESTELI framework offers structure for analysis, but the study remains descriptive and exploratory. Some of the conclusions read as prescriptive and definitive when they should be framed more cautiously. In addition, the ecological dimension of shortages was not explored, despite its importance in antibiotic manufacturing and the potential link to antimicrobial resistance in the environment.  

**Response: **We have modified the conclusion section. Due to the lack of evidence from the systematic review on the ecological impact of antibiotic shortages we did not explore this domain and allowed for the study participants to bring this up in the conversation. Additionally, our participants did not provide any information on this element.  

**Comment: **Another issue is novelty. The authors recently published a systematic review on antibiotic shortages, and the present work risks being seen as incremental rather than substantially new. The overlap between the two outputs should be made very clear.  

**Response: **The systematic review and this study are both part of the same research project. Hence our intention through these interviews were to collect more in-depth data.  

**Comment: **On balance, I believe the paper has potential value as a situational analysis of supply chain perspectives, but in its current form it does not make a sufficiently strong or novel contribution to merit indexing.  

**Response: **We have modified the paper to say that the aim is to do a situational analysis using key informant interviews with actors actively involved nationally and globally in antibiotic supply chain management to explore the main drivers for current shortages, existing mitigation strategies undertaken in different regions and potential barriers in their implementation.

# Reviewer report: Reviewer response for version 1

**DOI:** 10.21956/wellcomeopenres.26790.r127714

**Published:** 2025-08-27

**Authors:** Enrico Baraldi

**Affiliations:** 1 Uppsala University, uppsala, Sweden

This is a timely and highly relevant study, which addresses a key challenge for healthcare systems around the world, namely antibiotic shortages. The paper offers a broad and variegated picture of the issue at hand, with the merit of representing several countries with a global spread. The authors followed a systematic approach and offer a clear narrative, building on sufficient first-hand empirical materials. They also rely on a relevant conceptual framing around PESTELI (with risk however of ending up being a bit superficial in the results that can be reported, due to the many dimensions to be covered within this framework). Previous works in this area are generally identified and accounted for.

 This paper has the potential to offer an important contribution to the topic of antibiotic shortages, but it also presents some issues that would need to be addressed to make it more clear and impactful. Below here comes a set of suggestions for developing and improving the study.

 Suggestions for improvements:

 -Despite a brief mention on page 2 (“explore the main drivers for current shortages and existing mitigation strategies”), the purpose and focus of the paper is not entirely clear: is it about categorizing (around PESTELI) the causes of shortages, potential policies against them or barriers to introducing these policies, only some of these elements or all of them?

 -While the application of the PESTELI framework certainly makes sense, the authors should explain why they have chosen it, for instance by mentioning the fact that there are many different causes of shortages and potential responses to address them, which include elements of the dimensions included in the PESTELI framework. An alternative approach of focussing for instance only on selected dimensions, such as the economic or political ones, could have also been weighed against the approach chosen in terms of pros and cons.

 -There is a clear unbalance among sampled experts towards South Africa. Can you please explain why as many as 8 out of 16 respondents were selected from this country, as well as which implications this potential bias have on the study’s results?

 -The logic for sampling respondents from the five selecting countries (+EU level), which are then even used as mini-cases in table 2, needs to be explained. Does it have to do with differences in healthcare systems, national income levels, policies to address antibiotic shortages?

 -It is clearly important to consider the geographic spread of respondents, as different countries face different challenges in improving access to antibiotics and different specific causes of shortages, suggesting consequently also different interventions for specific countries. While the geographic origins of respondents is accounted for in the paper, it would be equally important to know which sector the various experts represent, for instance, industry, healthcare professionals, policy/public authorities and academia, because stakeholders from these different sectors perceive the problem of shortages from different perspectives and have indeed different interests, motivations and available strategies to deal with shortages.

 -It would be useful to know what type of antibiotic shortages the paper focuses on, considering in particular short vs long-term and deregistration vs lack of registration. In fact, different types of shortages (or lack of access) have different causes and can be addressed through different policies (see [Reference 1]).

 -The structure of the results section is not easy to grasp, and is not explained at its very start, making it difficult for readers to follow the logic of arguments, except after the PESTELI framework is explicitly applied from the bottom of page 5. Readability would increase with a clear explanation of the structure and contents at the very start of the result section. Moreover, it is not clear why the section starts with discussing the differences between the five countries in focus, followed by causes of shortages at a general level. Probably, inverting these two first sub-sections would make more sense, as the logic flow would go from global items to country-specific ones. Moreover, as it stands now, Table 2, including details about the five selected countries provides many details which are actually already about policies to address and handle shortages or lack of access, making further sense to present these after the sub-section about causes of shortages.

 -It is not clear why certain causes of shortages shown on Figure 1 have been attributed to a specific dimension of PESTELI. For instance, why is “transporting and assembling to a final product” part of the sociological/ecological area? This factor seems more a technological or industry issue. Similarly, the “mismatch demand-supply” seems more an economic or industry issue than a sociological/ecological one: for instance, a small country’s yearly demand of a specific antibiotic may be smaller in volume than the size of a production batch, requiring several years before that country consumes the allocated volumes. “Hoarding behaviour” would deserve in my view a special place among the sociological dimensions on Figure 1, as it is also mentioned in the text on page 8/9.

 -It is not clear if the various subsections featuring the PESTELI elements concern causes of shortages or policies to address them, and to what extend the differences between the 5 case countries are considered. In fact, there is some inconsistency and lack of balance as to how much of causes, policies and country differences are considered under the various PESTELI sub-headings. Some of these sub-headings even considers consequences of shortages (e.g., the sociological one), an element which does not seem to be part of the purpose/scope of the paper. All in all, a more consistent structure and balance in the space dedicated to causes, followed by policies and country differences would make it easier to follow these parts of the results section.

 -Legal elements appear in the headings of both the first and a subsequent PESTELI sub-heading in the result section. Are the latter “legal factors” as opposed to “legal frameworks” referring to different issues?

 -As for “Industry factors”, the starting sentence “By and large, the role of industry is absent as an active partner in working to mitigate shortages.” may apply to South Africa and India, but certainly not to Sweden and possibly not to UK and USA, where industry is mandated to at the very least report upcoming shortages.

 -It is not clear which role is played by Figure 2 and the very last sub-section of the result section (Mitigation strategies…). The previous sub-sections have already presented various strategies and policies, classified according to PESTELI. So, why do we need an additional sub-section about policies at the very end of the result section?

 -Discussion: It is a bit surprising that a key point made at the start of this section is about a very specific and ambitious policy intervention such as “pooled procurement”, which was basically not mentioned in the result section. If it is so important, it should have been presented among the results, ideally as a legal and economic item according to the PESTELI framework.

 -Conclusions: this section is rather short and general, which does not match the richness and depth of the study. Ideally, this section could stress the key findings of the study and what makes it particularly useful, namely its unique contribution to the issue of antibiotic shortages.

 Minor issues:

 -The paper would benefit from proof-reading as it includes a few grammatical inconsistencies that should be amended. For instance: “a global shortages crises” (bottom of page 2); or “Current nationally available data resources on status of or respond to shortages” (heading of Table 2); “…whilst government is invested in supporting…” (bottom of page 5); or “…may be therefore have its limitations” (bottom of page 8).

 -As for Sweden, Table 2 should mention in the right-most column that, similarly to UK and US, the Swedish Medical Product Agency runs a constantly updated list of drugs in shortages, and for antibiotics they also gather an expert group of ID specialists to propose alternative antibiotics in case of shortages.

 -Again for Sweden, the following sentence is not correct: “Swedish model aims to generate additional manufacturer revenue to maintain the supply chain of five selected antimicrobials” (page 8), because this new payment model aimed to stimulate the registration in Sweden of 5 new antibiotics still protected by exclusivity, not to improve or maintain their supply chain.

 I hope these comments can help the author further develop their paper.

 References:

 [Reference 1]

Is the work clearly and accurately presented and does it cite the current literature?

Yes

If applicable, is the statistical analysis and its interpretation appropriate?

Not applicable

Are all the source data underlying the results available to ensure full reproducibility?

Yes

Is the study design appropriate and is the work technically sound?

Yes

Are the conclusions drawn adequately supported by the results?

Partly

Are sufficient details of methods and analysis provided to allow replication by others?

Yes

Reviewer Expertise:

Industrial engineering and management, marketing, industrial and innovation policy

I confirm that I have read this submission and believe that I have an appropriate level of expertise to confirm that it is of an acceptable scientific standard, however I have significant reservations, as outlined above.

## References

[1] : The multifaceted nature of lack of access to antibiotics: types of shortage and specific causes, consequences, and solutions. *Clinical Microbiology and Infection* .2025;31(3) : 10.1016/j.cmi.2024.11.012 333-338 10.1016/j.cmi.2024.11.012 39536825

# Response

**Published:** 2025-11-25

**Authors:** ESMITA CHARANI

**Affiliations:** Medicine, University of Cape Town, Cape Town, Western Cape, South Africa

**Comment:** This is a timely and highly relevant study, which addresses a key challenge for healthcare systems around the world, namely antibiotic shortages. The paper offers a broad and variegated picture of the issue at hand, with the merit of representing several countries with a global spread. The authors followed a systematic approach and offer a clear narrative, building on sufficient first-hand empirical materials. They also rely on a relevant conceptual framing around PESTELI (with risk however of ending up being a bit superficial in the results that can be reported, due to the many dimensions to be covered within this framework). Previous works in this area are generally identified and accounted for.  

**Response: **The authors thank the reviewer for highlighting the relevance of the study.  

**Comment:** This paper has the potential to offer an important contribution to the topic of antibiotic shortages, but it also presents some issues that would need to be addressed to make it clearer and more impactful. Below here comes a set of suggestions for developing and improving the study.

**Response: **We have responded point by point to the comments and have made the recommended changes to the manuscript.  

**Comment:** Suggestions for improvements:

 -Despite a brief mention on page 2 (“explore the main drivers for current shortages and existing mitigation strategies”), the purpose and focus of the paper is not entirely clear: is it about categorizing (around PESTELI) the causes of shortages, potential policies against them or barriers to introducing these policies, only some of these elements or all of them? **Response: ** The aim of the study was to conduct a situational analysis using key informant interviews with actors actively involved, nationally and globally, in antibiotic supply chain management to explore the main drivers for current shortages, existing mitigation strategies undertaken in different regions and potential barriers in their implementation. We have added this as the aim consistently across the manuscript.  

**Comment:** While the application of the PESTELI framework certainly makes sense, the authors should explain why they have chosen it, for instance by mentioning the fact that there are many different causes of shortages and potential responses to address them, which include elements of the dimensions included in the PESTELI framework. An alternative approach of focussing for instance only on selected dimensions, such as the economic or political ones, could have also been weighed against the approach chosen in terms of pros and cons.  

**Response: ** We have further clarified why the PESTELI framework was used in lines 171-176.  

**Comment:** There is a clear unbalance among sampled experts towards South Africa. Can you please explain why as many as 8 out of 16 respondents were selected from this country, as well as which implications this potential bias have on the study’s results?

**Response: **Participation in the interview was voluntary. We reached out to stakeholders in South Africa, Europe, India and the USA and interviewed participants who consented. We have discussed the limitation this poses to the study.  

**Comment: **The logic for sampling respondents from the five selecting countries (+EU level), which are then even used as mini-cases in table 2, needs to be explained. Does it have to do with differences in healthcare systems, national income levels, policies to address antibiotic shortages?  

**Response: **Our intention was to include perspectives and experiences from different income setting countries/regions. This follows on from our systematic review, referenced in our manuscript which highlights that the published evidence from shortages comes mainly from high income countries.  

**Comment:** It is clearly important to consider the geographic spread of respondents, as different countries face different challenges in improving access to antibiotics and different specific causes of shortages, suggesting consequently also different interventions for specific countries. While the geographic origins of respondents is accounted for in the paper, it would be equally important to know which sector the various experts represent, for instance, industry, healthcare professionals, policy/public authorities and academia, because stakeholders from these different sectors perceive the problem of shortages from different perspectives and have indeed different interests, motivations and available strategies to deal with shortages.  

**Response: **Thank you for this comment. We have added another column to table 1 to include the sector where the interview participants work.  

**Comment:** It would be useful to know what type of antibiotic shortages the paper focuses on, considering in particular short vs long-term and deregistration vs lack of registration. In fact, different types of shortages (or lack of access) have different causes and can be addressed through different policies (see [Reference 1]).  

**Response: **We discussed in our interview guide shortages of licensed antibiotics. We have clarified this.  

**Comment:** The structure of the results section is not easy to grasp, and is not explained at its very start, making it difficult for readers to follow the logic of arguments, except after the PESTELI framework is explicitly applied from the bottom of page 5. Readability would increase with a clear explanation of the structure and contents at the very start of the result section. Moreover, it is not clear why the section starts with discussing the differences between the five countries in focus, followed by causes of shortages at a general level. Probably, inverting these two first sub-sections would make more sense, as the logic flow would go from global items to country-specific ones. Moreover, as it stands now, Table 2, including details about the five selected countries provides many details which are actually already about policies to address and handle shortages or lack of access, making further sense to present these after the sub-section about causes of shortages.   

**Response: **We have restructured the manuscript and added more detail, including justification for PESTELI, in line with the reviewer’s comment.   

**Comment:** It is not clear why certain causes of shortages shown on Figure 1 have been attributed to a specific dimension of PESTELI. For instance, why is “transporting and assembling to a final product” part of the sociological/ecological area? This factor seems more a technological or industry issue. Similarly, the “mismatch demand-supply” seems more an economic or industry issue than a sociological/ecological one: for instance, a small country’s yearly demand of a specific antibiotic may be smaller in volume than the size of a production batch, requiring several years before that country consumes the allocated volumes. “Hoarding behaviour” would deserve in my view a special place among the sociological dimensions on Figure 1, as it is also mentioned in the text on page 8/9.  

**Response: **We have modified figure 1 to address this comment.    

**Comment:** It is not clear if the various subsections featuring the PESTELI elements concern causes of shortages or policies to address them, and to what extend the differences between the 5 case countries are considered. In fact, there is some inconsistency and lack of balance as to how much of causes, policies and country differences are considered under the various PESTELI sub-headings. Some of these sub-headings even considers consequences of shortages (e.g., the sociological one), an element which does not seem to be part of the purpose/scope of the paper. All in all, a more consistent structure and balance in the space dedicated to causes, followed by policies and country differences would make it easier to follow these parts of the results section.  

**Response: **We acknowledge this variation in our findings and we interpret it as the complexity and diversity of issues that impact of antibiotic shortages in different countries.   Comment Legal elements appear in the headings of both the first and a subsequent PESTELI sub-heading in the result section. Are the latter “legal factors” as opposed to “legal frameworks” referring to different issues?   Response We have edited the sub heading to address this comment.  

**Comment:** As for “Industry factors”, the starting sentence “By and large, the role of industry is absent as an active partner in working to mitigate shortages.” may apply to South Africa and India, but certainly not to Sweden and possibly not to UK and USA, where industry is mandated to at the very least report upcoming shortages.  

**Response: **We made this point based on the data we collected through our interviews. Since there was a lack of participation from the pharmaceutical industries, we could not explore these further.  

**Comment:** It is not clear which role is played by Figure 2 and the very last sub-section of the result section (Mitigation strategies…). The previous sub-sections have already presented various strategies and policies, classified according to PESTELI. So, why do we need an additional sub-section about policies at the very end of the result section?  

**Response: **In addition to the mitigation strategies elaborated under the various elements of PESTELI in the results, the interview participants also provided some potential strategies to better handle situations of antibiotic shortages which are shown in Figure 2.    

**Comment:** Discussion: It is a bit surprising that a key point made at the start of this section is about a very specific and ambitious policy intervention such as “pooled procurement”, which was basically not mentioned in the result section. If it is so important, it should have been presented among the results, ideally as a legal and economic item according to the PESTELI framework.

**Response: **We have discussed about pooled procurement under **Results** in lines 238-241.  

**Comment:** Conclusions: this section is rather short and general, which does not match the richness and depth of the study. Ideally, this section could stress the key findings of the study and what makes it particularly useful, namely its unique contribution to the issue of antibiotic shortages.  

**Response: **The conclusion section has been modified in response to this comment.  

**Comment:** Minor issues:

 -The paper would benefit from proof-reading as it includes a few grammatical inconsistencies that should be amended. For instance: “a global shortages crises” (bottom of page 2); or “Current nationally available data resources on status of or respond to shortages” (heading of Table 2); “…whilst government is invested in supporting…” (bottom of page 5); or “…may be therefore have its limitations” (bottom of page 8).  

**Response: **The authors thank the reviewer for pointing these errors. We have corrected them in the manuscript.  

**Comment:** As for Sweden, Table 2 should mention in the right-most column that, similarly to UK and US, the Swedish Medical Product Agency runs a constantly updated list of drugs in shortages, and for antibiotics they also gather an expert group of ID specialists to propose alternative antibiotics in case of shortages.  

**Response: **We have consulted the Swedish Medical Product Agency website and found a search engine that provides Information about reported medicine shortages. We have provided information regarding this in table 3. We could not find any details on the expert group of ID physicians.  

**Comment:** Again, for Sweden, the following sentence is not correct: “Swedish model aims to generate additional manufacturer revenue to maintain the supply chain of five selected antimicrobials” (page 8), because this new payment model aimed to stimulate the registration in Sweden of 5 new antibiotics still protected by exclusivity, not to improve or maintain their supply chain.

**Response: **This is the quote verbatim from a participant. We cannot change it. We have provided context for this, in line with the advice from this comment.
